# Global knowledge mapping of receptor activator of nuclear factor kappa-B ligand in osteoporotic fractures: a bibliometric analysis (2001–2024)

**DOI:** 10.3389/fmolb.2025.1545109

**Published:** 2025-03-26

**Authors:** Shuai Lu, Huaishuang Shen, Minjuan Li, Yiming Luo, Hao Sun, Xian Zhao, Jianming Chen, Ruifeng Bai, Pengli Han, Yejun Zha, Xieyuan Jiang

**Affiliations:** ^1^ Department of Orthopedic Trauma, Beijing Jishuitan Hospital, Capital Medical University, Beijing, China; ^2^ Beijing Research Institute of Traumatology and Orthopaedics, Beijing, China; ^3^ Department of Orthopaedic Surgery, First Affiliated Hospital of Soochow University, Suzhou, China; ^4^ Geriatric Orthopedic, Shenzhen Pingle Orthopedic Hospital (Shenzhen Pingshan Traditional Chinese Medicine Hospital), Shenzhen, China; ^5^ Department of orthopedic, People’s Hospital of Lingcheng District, Dezhou, China; ^6^ Department of Clinical Laboratory, Beijing Jishuitan Hospital, Capital Medical University, Beijing, China; ^7^ Pharmaceutical Department, Zhengzhou Central Hospital Affiliated to Zhengzhou University, Zhengzhou, China

**Keywords:** osteoporotic fractures, RANKL, bone metabolism, osteoclast differentiation, bibliometric analysis

## Abstract

**Background:**

Receptor activator of nuclear factor kappa-B ligand (RANKL) plays a critical role in bone metabolism and the pathogenesis of osteoporotic fractures. This study aims to conduct a bibliometric analysis of global research pertaining to RANKL and osteoporotic fractures to identify key trends, influential studies, and collaborative networks.

**Methods:**

A literature search was conducted to identify articles found in the Web of Science Core Collection database regarding RANKL and osteoporotic fractures from 2001 to 2024. A bibliometric analysis was performed using VOSviewer, CiteSpace, and R 4.3.3 for the publication volume, country and institution contributions, journal impact, author influence, and research hotspots.

**Results:**

A total of 214 articles were analyzed. Publication rates have steadily increased, with a peak of 21 papers in 2020. The U.S., China, and South Korea were the top contributing countries, and leading institutions included Harvard University and Dankook University. The *Journal of Bone and Mineral Research*, *Osteoporosis International*, and *Bone* were the journals of highest impact. At the level of authors, Heiss–Christian published the highest number and Christiansen–Claus had the strongest citation impact (1,368 citations). Research evolved from basic biological mechanisms (2001–2010) through clinical applications (2011–2017) to recent renewed interest in fundamental RANKL biology (2018–2024). Key research hotspots included postmenopausal osteoporosis, bone mineral density, and osteoclast differentiation, with emerging focus on RANKL’s role beyond skeletal metabolism.

**Conclusion:**

This bibliometric analysis provides a comprehensive overview of RANKL research in osteoporotic fractures, highlighting key priorities for future investigation. Future studies should prioritize understanding RANKL’s broader physiological roles, developing better predictive markers, and optimizing personalized treatment strategies.

## Introduction

Osteoporotic fractures are acknowledged as a significant public health issue and represent one of the primary causes of morbidity and mortality on a global scale (Shen et al., 2022). They impose a substantial burden on healthcare systems and adversely affect the quality of life for affected individuals (Kumar et al., 2021). Projections indicate that by 2050, the global incidence of these fractures is expected to increase by 310% for men and 240% for women when compared to levels observed in 1990, a trend primarily attributable to aging populations (Wu et al., 2021). The underlying pathogenesis is characterized by an imbalance in bone remodeling, which is marked by excessive bone resorption that surpasses bone formation, ultimately leading to a reduction in bone mass and the deterioration of bone microarchitecture (Armas and Recker, 2012). This dysregulation is governed by intricate cellular and molecular pathways, with osteoclasts playing a crucial role in heightened bone resorption (Chen et al., 2018). At the molecular level, various signaling molecules and cytokines modulate this process, with the RANKL/RANK/OPG axis emerging as a central mediator (Zhang et al., 2022). The development of fragility fractures in osteoporosis is further influenced by a range of risk factors, including age, hormonal status, mechanical loading, and inflammatory mediators, all of which can affect the expression and activity of these molecular regulators (Khosla and Monroe, 2018). A comprehensive understanding of these pathogenic mechanisms is increasingly essential for early diagnosis and intervention, as timely treatment can impede disease progression and mitigate the risk of fractures (LeBoff et al., 2022).

Receptor Activator of Nuclear Factor Kappa-B Ligand (RANKL) is integral to the pathophysiology of osteoporosis and its related fractures (Sakai, 2023). It belongs to the tumor necrosis factor family. RANKL controls how osteoclasts develop and function (Yao et al., 2021). The dysregulation of the RANKL/RANK/osteoprotegerin (OPG) axis, which regulates the balance between osteoclastogenesis and osteoblastogenesis, represents a significant mechanism underlying bone remodeling. An imbalance within this axis results in increased osteoclastic bone resorption and diminished osteoblastic bone formation, both of which are hallmark characteristics of osteoporosis (Zhang et al., 2022). Over the past 2 decades, considerable efforts have been directed toward elucidating the complex interplay between RANKL and osteoporotic fractures (Matsumoto and Endo, 2021; Kim B.-J. et al., 2020). This research has significantly advanced the understanding of bone metabolism and has facilitated the development of novel therapeutic strategies.

Bibliometric analysis serves as a significant methodological approach for evaluating the impact and trends within the existing body of scientific literature, offering a quantitative overview of research developments. (Ball, 2017). This analysis elucidates the evolution of specific research domains, identifies key contributors and collaborative efforts, and indicates potential emerging research topics. While several bibliometric studies have examined osteoporosis research broadly (Li et al., 2023; Kim and Lee, 2024; Rong et al., 2024), they have focused primarily on general treatment approaches, clinical aspects in specific populations, or particular cellular mechanisms. Despite RANKL’s central role in osteoporosis pathophysiology and its emergence as a therapeutic target, there has not been a comprehensive bibliometric analysis specifically examining the intersection of RANKL and osteoporotic fractures. This gap is particularly notable given the rapid evolution of RANKL research over the past 2 decades, from initial molecular characterization to therapeutic applications. Therefore, this study aims to conduct a comprehensive bibliometric analysis of global research pertaining to RANKL and osteoporotic fractures. Conducting such an analysis would be highly advantageous for understanding the current landscape of this critical field, particularly in identifying knowledge gaps and delineating future research needs.

## Materials and methods

### Data source and search strategy

A bibliometric analysis of publications associated with RANKL and osteoporotic fractures was performed through the Web of Science Core Collection (WoSCC). We searched using the following query: TS=((“Osteoporotic Fracture*”) AND (“Receptor Activator of Nuclear Factor κB Ligand” OR “RANKL”)). The literature search conducted on 29 May 2024 was based on a period of 2001–2024. The data that we retrieved for each publication included the title, author, institution, country/region, number of publications and citations, keywords and journal name.

### Inclusion and exclusion criteria

We applied the following inclusion criteria (Shen et al., 2022): original research articles focusing on RANKL in the context of osteoporotic fractures (Kumar et al., 2021); articles with available abstracts and citations (Wu et al., 2021); articles with complete bibliometric information. Exclusion criteria encompassed (Shen et al., 2022): articles mentioned RANKL and osteoporotic fractures but with irrelevant topics.

### Data analysis and visualization

From the retrieved literature records, we extracted relevant data and calculated bibliometric indicators using Microsoft Excel. We employed three powerful bibliometric analysis tools for comprehensive analysis of the academic data. VOSviewer (version 1.6.20) (Van Eck and Waltman, 2010) was used for spatial analysis of relationships between institutions, authors, and citations. This tool allowed us to visualize collaboration networks and co-citation patterns; CiteSpace (version 6.3. R1) (Chen, 2006) was utilized for keyword burst detection and revealing emerging trends in the field. We used pathfinder network scaling and pruning to simplify the network structure. Set the parameters to time slicing: January 2001 - September 2024, node types: keywords. When the node is a keyword: threshold (top N per segment) = 5, pruning = pathfinder + pruning merge network. Based on the parameter settings for each node, perform a visual analysis to generate a keyword timeline graph; The R 4.3.3 (Aria and Cuccurullo, 2017) was employed for additional visualizations and analyses, including country collaboration networks and journal impact evaluations.

We evaluated the academic influence of individual researchers and journals based on the H-index (Bertoli-Barsotti and Lando, 2017; Hirsch, 2005). It is defined as the maximum number of papers with number of citations that each have at least h citations. The G-index and M-index were also computed for a more comprehensive assessment of impact (Schreiber, 2010; Novak et al., 2011). While the G-index (one of the variants of H-index) gives preferential weight to the highly cited articles, the M-index is similar to H-index, but it is further divided by the number of years since the first published paper, accounts for the duration of a research career. The journal impact factors and quartile rankings were obtained from the 2023 Journal Citation Reports (JCR) (Gould, 2023). A journal’s IF represents the yearly average number of citations to recent articles published in that journal, while the quartile ranking (Q1-Q4) indicates its relative standing within its subject category, with Q1 representing the top 25% of journals (Salisbury, 2020).

## Results

### Overview of publication output

**FIGURE 1:**
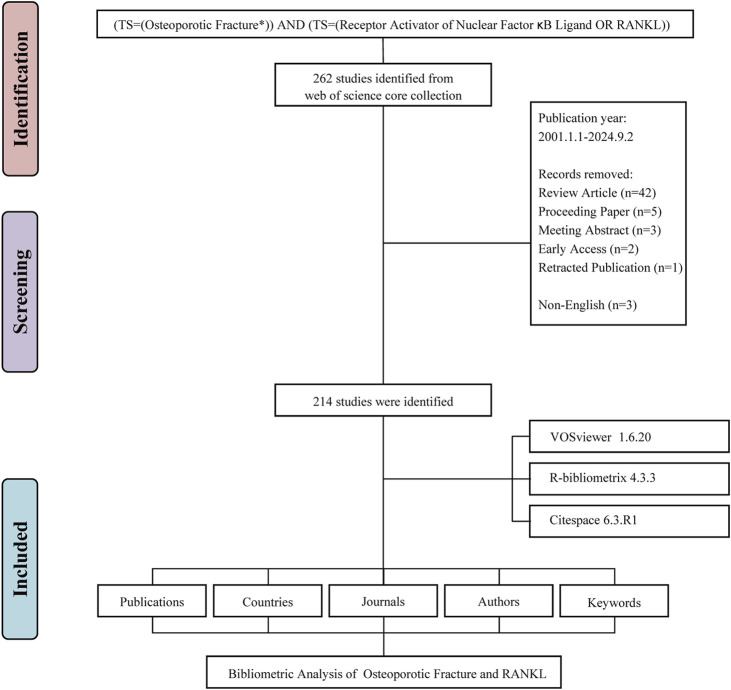
Flowchart of the literature screening process.

**FIGURE 2:**
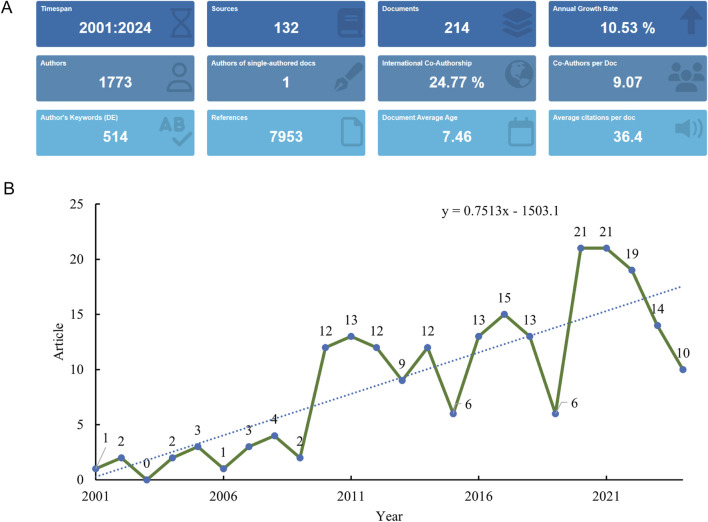
Analysis of general information. **(A)** Summary information of the included studies. **(B)** Annual number of publications.

The systematic literature search initially identified 262 potentially relevant publications addressing RANKL in the context of osteoporotic fractures spanning from 1 January 2001 to 2 September 2024. After applying exclusion criteria, we removed 48 records that did not meet the inclusion criteria: 42 review articles, 5 proceeding papers, 3 meeting abstracts, 2 early access publications, 1 retracted publication, and 3 non-English articles. The remaining 214 original research articles were included in the final analysis (Figure 1). These articles represent the collective efforts of 1,773 authors. The depth of commitment to this research area is shown through the 7,953 references cited as part of these publications, attesting to the robust knowledge base underpinning this field (Figure 2A). The annual publications growth rate from 2001 to 2024 was determined, yielding a 10.53% compound annual growth rate (CAGR), which highlights the excellent growth of research output over the last 2 decades. This growth rate indicates a rising scientific interest in RANKL as a target for osteoporotic fractures. The overall publication output presented an upward trend per year with some fluctuations over the years in the study period. It started with only 2 articles published in the year 2001 for this area of research. The numbers subsequently increased every year, peaking in 2019 with 18 publications (Figure 2B).

### Geographical distribution of research

**FIGURE 3:**
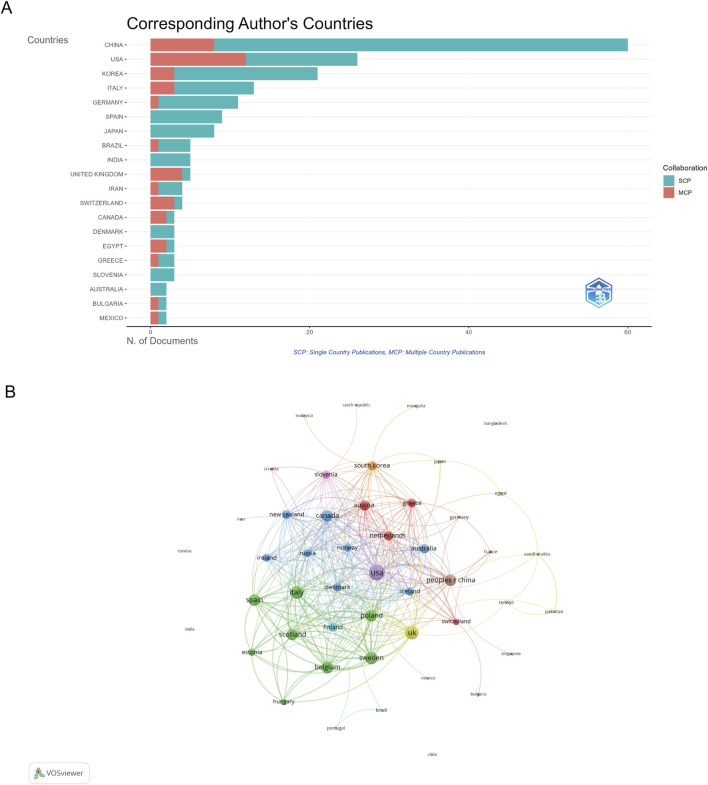
Analysis of countries. **(A)** Distribution of corresponding author’s publications by country. **(B)** Visualization map depicting the collaboration among different countries.

The identified publications came from 115 countries, with China leading in the number of studies (60 publications), constituting 28% of all documents. Other top contributors included the United States (26 publications), Korea (21 publications), Italy (13 publications), and Germany (11 publications) (Figure 3A; Supplementary Table S1). A detailed analysis of country-specific contributions and their citation impacts can be found in Supplementary Table S1, which shows that despite China having the highest number of articles, Denmark, Canada, and the United States demonstrated the highest average citations per paper, at 84.3, 72.0, and 69.5, respectively.

The collaboration among countries was visualized using VOSviewer (Figure 3B). The visualization map of international collaboration networks revealed varying degrees of connection strength among 47 countries. Analysis of total link strength showed that the United States maintained the strongest collaborative ties with a total link strength of 66, indicating the intensity and frequency of its research partnerships. The United Kingdom followed with a link strength of 48, while Italy demonstrated robust international research connections with a link strength of 47.

### Journal analysis and publication patterns

The bibliometric indicators of high-impact journals in the field are shown in Table S2. The dataset comprised of 214 articles published in 132 journals conveying a wider spread of research findings. As detailed in Table S2, 10 articles (4.67% of total publications) with 741 citations (9.51% of total citations) were published in the *Journal of Bone and Mineral Research*, making it by far the dominant journal in the field. *Osteoporosis International* was next with 9 papers and 394 citations, while *Bone* had 8 papers with 491 citations.

When considering the impact factor of journals, as reported in Supplementary Table S2 from the 2023 Journal Citation Reports, *Bone Research* led with an impact factor of 14.3, followed by *Biomaterials* (IF: 12.8) and *Cell Death & Disease* (IF: 8.1). However, it is worth noting that these high-impact journals published fewer articles in the dataset compared to the specialized bone and mineral journals.

**FIGURE 4:**
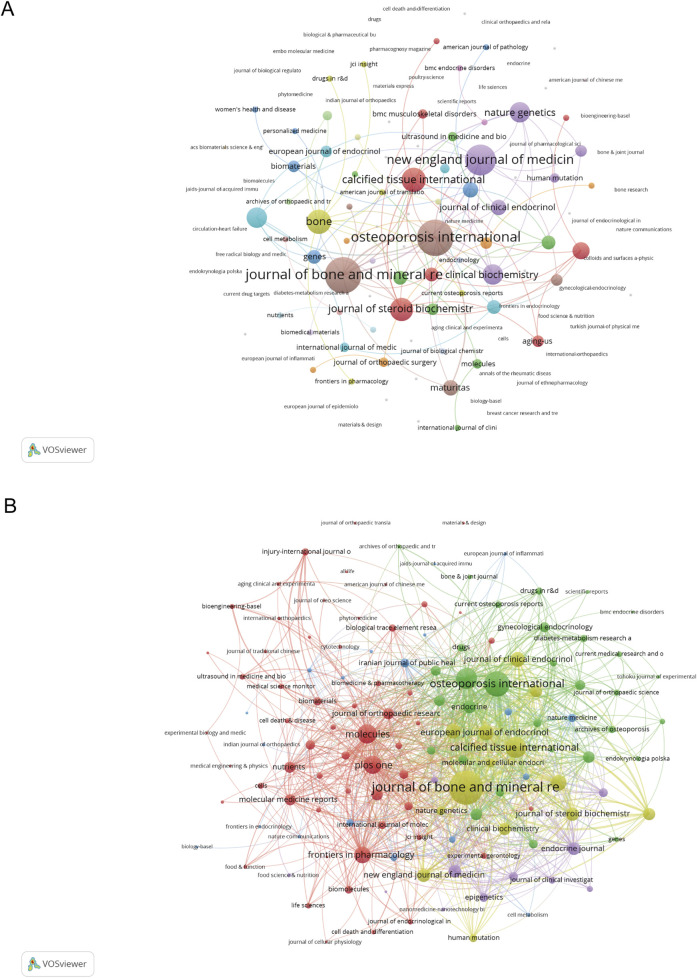
Analysis of journals. **(A)** Co-occurrence Network of Journals. **(B)** Coupling Network of Journals.

The co-occurrence networks of journals contain 132 with at least 1 occurrence (Figure 4A). The three key journals with the highest total link strength in co-occurrence networks were *Journal of bone and mineral research* (Novak et al., 2011), *Osteoporosis international* (Novak et al., 2011), and *New England journal of medicine* (Van Eck and Waltman, 2010). The network visualization of journal co-citation patterns appears in Figure 4B. The coupling network analysis of these same journals revealed substantially stronger connections, with the *Journal of Bone and Mineral Research* showing a dominant link strength of 1,024, followed by *Osteoporosis International* (592) and *Bone* (436), indicating robust citation relationships among these key publications.

### Author productivity and collaboration

Our analysis found 1,773 authors across 214 publications. Each paper had an average of 9.07 authors. This high number suggests strong research collaboration. Supplementary Table S3 provides the publication and citation profiles of high-impact authors within the area.

The most productive author in terms of publication count was Heiss, Christian, with 6 publications, closely followed by Alt, Volker and El Khassawna, Thaqif, each with 5 publications. However, when considering citation impact, a different picture emerged. As shown in Supplementary Table S3, Christiansen, Claus, despite having only 3 publications in the dataset, received the highest number of citations (1,368), resulting in an average of 456 citations per paper.

The author co-citation analysis revealed several distinct clusters of researchers, suggesting the existence of different sub-fields or research focuses within the broader area of RANKL and osteoporotic fractures. One prominent cluster centered around researchers focusing on genetic aspects of osteoporosis, including Estrada, K. and Richards, J.B. Another cluster comprised researchers primarily investigating the clinical applications of RANKL inhibitors, including Cummings, S.R. and McClung, M.R.

**FIGURE 5:**
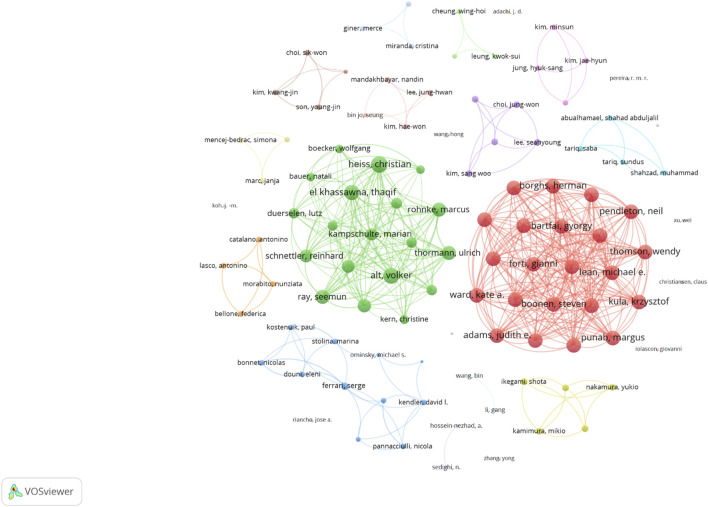
Visualization map depicting the collaboration among different authors.

The collaboration network analysis showed a complex web of co-authorships, with several key researchers acting as bridges between different research groups (Figure 5). The author collaboration network, comprising 106 researchers with at minimum two articles, identified Heiss, Christian as the most well-connected researcher with a link strength of 53. Alt, Volker and El Khassawna, Thaqif followed closely with link strengths of 47 and 46, respectively, indicating strong collaborative relationships among these key researchers in the field.

### Institutional contributions and collaborations

**FIGURE 6:**
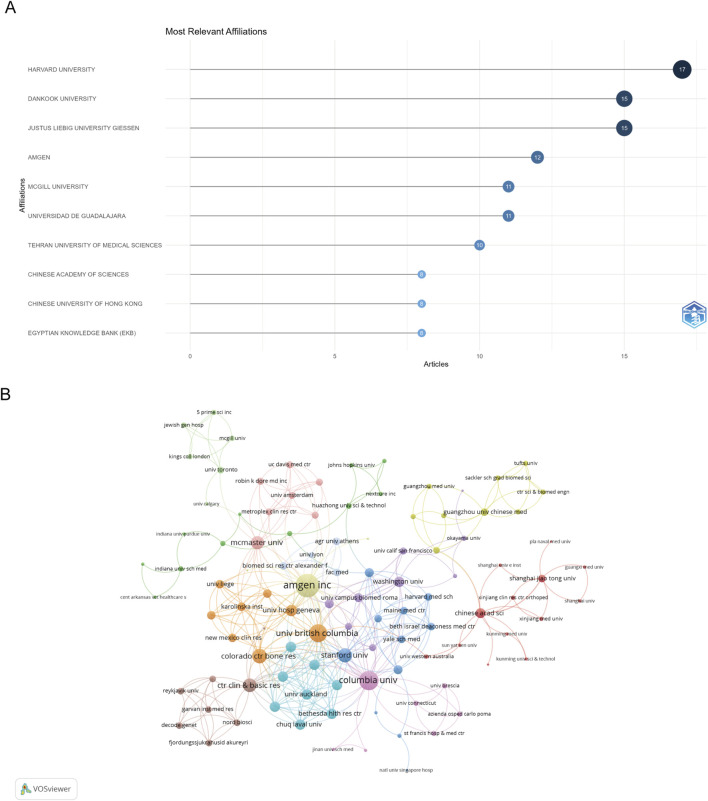
Analysis of institutions. **(A)** Top ten institutions by article count and rank. **(B)** Visualization map depicting the collaboration among different institutions.

A total of 996 institutions contributed to the research output in the dataset. Figure 6A displays the top ten institutions by article count and rank. Harvard University emerged as the most productive institution with 17 publications, followed closely by Dankook University and Justus Liebig University Giessen, both with 15 publications each.

The collaboration network analysis revealed dense interconnections among institutions, particularly those within the same country (Figure 6B). The institutional collaboration analysis identified 487 institutions with notable international research partnerships. Amgen Inc. emerged as the most strongly connected institution with a link strength of 47, while Columbia University and the University of British Columbia demonstrated significant collaborative ties with link strengths of 34 and 30, respectively. This pattern highlights the important role of both industry and academic institutions in fostering research collaborations.

### Most cited articles and their impact

The 50 most highly cited publications on RANKL and osteoporotic fractures are listed in Supplementary Table S4, and all represented important contributions to the field. Out of all articles in the dataset, the most cited article was “Genome-wide meta-analysis identifies 56 bone mineral density loci and reveals 14 loci associated with risk of fracture” by Estrada et al., published in *Nature Genetics* in 2012 (26). As detailed in Supplementary Table S4, this seminal paper received 905 citations up to the present time, highlighting its significant impact on the field. The complete citation metrics and impact factors for all highly cited papers can be found in Supplementary Table S4, which provides a comprehensive overview of the field’s most influential research contributions. The second most cited article (434 citations), was “Multiple genetic loci for bone mineral density and fractures” by Styrkarsdottir et al., published in *The New England Journal of Medicine* in 2008 (27). The third most cited article, related to the topical area of interest, is “Receptor activator of NF-kappa B and osteoprotegerin expression by human microvascular endothelial cells, regulation by inflammatory cytokines, and role in human osteoclastogenesis.” Published in *The Journal of Biological Chemistry* in 2001 (28). Three of the 10 most cited papers investigated genetic factors related to bone mineral density and fracture risk, appearing in leading journals including *Nature Genetics* and *The New England Journal of Medicine*. Two of the papers explored the core biology of RANKL signaling, and three were more focused on clinical applications and treatment strategies. The other two papers evaluated the association of RANKL with different metabolic axes.

### Keyword analysis and research hotspots

**FIGURE 7:**
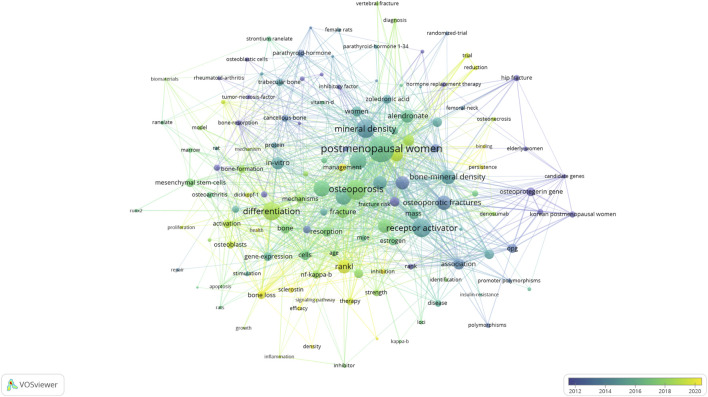
Visual analysis of keyword co-occurrence network analysis.

The keyword co-occurrence analysis, based on both author keywords and Keywords Plus, provided valuable insights into the main topics and trends in the field (Figure 7). After removing generic terms (e.g., “osteoporosis”, “RANKL”), we identified several key themes:

“Postmenopausal women” was the most common keyword (50 occurrences) and illustrates the commonality of this high-risk population for osteoporosis. Common keywords included many terms closely related to osteoporosis and fracture risk, with “bone mineral density (32 occurrences) and “fracture risk” (26 occurrences) being top mentioned–overall reflecting these concepts as a prominent theme by which to assess osteoporosis and the ability of individual to sustain a fracture. A few of the clusters were distinct based on the keyword co-occurrence network obtained. A cluster focused on clinical and epidemiological terms (e.g., “postmenopausal osteoporosis”, “fracture risk”, “bone mineral density”) about clinical and epidemiological terms, whereas the other was on molecular and cellular mechanisms (e.g., “osteoclast differentiation”, “NF-kappa B”, “signaling pathway”).

The temporal analysis of keywords using overlay visualization in VOSviewer revealed shifts in research focus over time. In the early years (2001–2010), they contained keywords mainly related to field of basic biology and mechanisms (e.g., “osteoclastogenesis”, “cytokines”). The second period (2011–2017) is marked by more clinical and therapeutic keywords (e.g., “denosumab”, “bisphosphonates”), while the last years (2018–2024) are enriched with more genetic and personalized medicine keywords (e.g., “genome-wide association”, “biomarkers”).

**FIGURE 8:**
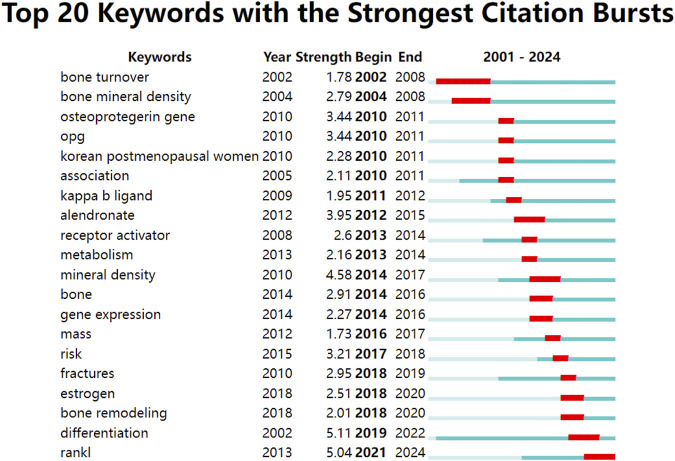
Top 20 keywords with the strongest citation bursts.

The burst detection analysis using CiteSpace identified several keywords with strong citation bursts, indicating topics that have received sudden attention from the research community. Notable bursts included “mineral density” (strength: 4.58, 2014–2017), “alendronate” (strength: 3.95, 2012–2015), and most recently, “RANKL” itself (strength: 5.04, 2021–2024), suggesting a renewed focus on the fundamental biology of RANKL in recent years (Figure 8).

## Discussion

The bibliometric analysis of RANKL and osteoporotic fracture research from 2001 to 2024 reveals significant growth with a 10.53% annual publication increase, evolving from basic biological processes to clinical applications and recently returning to fundamental RANKL biology. Key journals include *Journal of Bone and Mineral Research*, *Osteoporosis International*, and *Bone*, with high-impact publications also appearing in *Bone Research* (IF: 14.3) and *Biomaterials* (IF: 12.8), indicating broader scientific interest beyond osteoporosis.

The United States and China produce the most publications with high citation impact. Denmark and Canada show notable research quality through high average citations. Harvard University and UC San Francisco lead institutional contributions. Amgen’s partnerships demonstrate strong industry-academia collaboration in clinical research. Notable researchers include Christiansen, Claus, with 3 highly cited publications totaling 1,368 citations, and Ferrari, S., who significantly influenced genetic basis research.

The most influential studies range from Estrada et al.'s genome-wide meta-analysis (905 citations) identifying BMD-associated and fracture risk loci, to Styrkarsdottir et al.'s genetic research (434 citations), and Collin-Osdoby et al.'s work (328 citations) on RANKL’s role in microvascular endothelial cells (Estrada et al., 2012; Styrkarsdottir et al., 2008; Collin-Osdoby et al., 2001). The three most highly cited papers have made distinctive contributions to understanding different aspects of osteoporotic fractures and RANKL biology. Estrada et al.'s landmark genome-wide meta-analysis (905 citations) comprehensively identified 56 loci associated with bone mineral density (BMD) and discovered 14 loci specifically linked to fracture risk (Estrada et al., 2012). This study revolutionized the understanding of genetic susceptibility to osteoporosis by providing the first large-scale evidence that BMD-decreasing alleles directly influence fracture risk, establishing a genetic basis for developing targeted therapeutic approaches. Styrkarsdottir et al.'s seminal genetic research (434 citations) identified five regions containing genes that influence BMD and highlighted the ESR1/C6orf97, RANKL, OPG, and ZBTB40 regions as key regulators of bone mass, demonstrating how common genetic variants contribute to the risk of osteoporosis and fractures (Styrkarsdottir et al., 2008). This work was instrumental in establishing the polygenic nature of osteoporosis and identifying novel therapeutic targets. Collin-Osdoby et al.'s foundational study (328 citations) revealed the critical role of microvascular endothelial cells in expressing RANKL and osteoprotegerin, demonstrating how these cells respond to inflammatory cytokines and participate in osteoclastogenesis (Collin-Osdoby et al., 2001). This work provided crucial insights into the vascular-bone axis and how inflammatory conditions might influence bone metabolism through endothelial cell-mediated mechanisms, opening new avenues for therapeutic intervention in bone diseases. Other significant contributions include research on bone homeostasis, RANKL signaling mechanisms, denosumab treatment, and vitamin D regulation in bone metabolism, demonstrating the field’s comprehensive progression through international collaboration and interdisciplinary approaches.

### Research hotspots and trends

The keyword burst analysis reveals critical transitions in research focus that parallel major developments in bone biology and therapeutics. During this early time period (2001–2010), the high burst strengths in such keywords as “bone turnover” (burst strength: 1.78, 2002–2008) and “bone mineral density” (burst strength: 2.79, 2004–2008) demonstrate that the focus was on basic bone biology and the development of quantitative measures of bone health. This is consistent with the early seminal description during this time of the RANKL/RANK/OPG pathway and its function in bone turnover (Boyce and Xing, 2007; Hooshiar et al., 2022). These foundational studies were crucial for establishing the basic mechanisms that would later inform therapeutic strategies.

Keywords in the second time period (2011–2017) became more clinical and therapeutic-oriented. For example, the high burst for “alendronate” (burst strength: 3.95, 2012–2015) corresponds to a growing number of studies on bisphosphonates and their use relative to new therapies targeting RANKL (Gennari et al., 2020; Song et al., 2022). This burst coincided with the emergence of RANKL inhibition as a therapeutic strategy, sparking investigations into how these new biologics compared with established treatments (Estell and Rosen, 2021). During this time several landmark clinical trials on denosumab were published including the FREEDOM extension study which highlighted RANKL inhibition as a long-term effective and safe therapy (Bone et al., 2017). This shift from basic science to clinical applications reflects the successful translation of molecular insights into therapeutic interventions.

Continual presence of “mineral density” (burst strength: 4.58) at all years in the study indicates its key importance in osteoporosis diagnosis and management. Nonetheless, the more recent upsurge in “fractures” (burst strength: 2.95, 2018–2019) suggests an increased awareness of the inadequacy of bone mineral density as a uni-dimensional predictor of fracture risk. This evolution reflects growing recognition that fracture prevention requires understanding beyond bone density measurements alone, driving development of more sophisticated risk assessment tools like FRAX (Kanis et al., 2020). This trend suggests future therapeutic approaches may need to address multiple aspects of bone quality beyond density.

The final period (2018–2024) shows diverse research topics. “Differentiation” emerged as a key term (burst strength: 5.11, 2019–2022). This reflects growing interest in cellular mechanisms of bone remodeling. This could indicate an increasing awareness of the importance of osteocytes as potential orchestrators of bone metabolism, as previously reviewed by Bonewald (2011) (Bonewald, 2011). This could lead to development of more sophisticated therapeutic approaches that modulate cell fate decisions rather than simply inhibiting RANKL activity.

Interestingly, the recent top hit for “RANKL” (burst strength: 5.04, 2021–2024) suggests a wave of interest in its transcriptional biology. The comeback may be related to underscored novel roles for RANKL beyond bone metabolism, such as immune regulatory functions and possible pathogenic links to conditions such as cancer and cardiovascular diseases (Infante et al., 2019). The evolution highlighted in the articles, from basic biology to clinical applications and back to fundamental questions with new lenses, is a hallmark of the iterative process that is biomedical research. Moreover, it underscores the enduring relevance of RANKL as both a research subject and substantial topic nearly 20 years after the initial isolation of this protein.

The trends and hotspots identified in this bibliometric analysis of RANKL research in osteoporotic fractures should be considered within the broader context of advances in osteoporosis and bone biology research. While our study focused specifically on the role of RANKL, it is important to recognize that this pathway operates within a complex network of signaling molecules, cell types, and metabolic processes that collectively regulate bone health.

In the field of osteoblast biology, recent research has highlighted the critical role of Wnt signaling in regulating bone formation and osteoblast differentiation (Zhu et al., 2024). Studies have shown that targeting Wnt signaling pathways, such as through the use of sclerostin antibodies, can stimulate bone formation and improve bone mass in animal models and human clinical trials (Marini et al., 2023; Vasiliadis et al., 2022). These findings suggest that therapies aimed at enhancing osteoblast function may complement RANKL-targeted approaches in the prevention and treatment of osteoporosis.

Another important area of research in bone metabolism involves the role of parathyroid hormone (PTH) signaling. PTH plays a key role in regulating calcium homeostasis and has both anabolic and catabolic effects on bone depending on the mode of administration (Rendina-Ruedy and Rosen, 2022). Intermittent PTH therapy has been shown to stimulate bone formation and improve bone mineral density in osteoporotic patients, representing another promising therapeutic approach (Zhang and Song, 2020). Understanding the interplay between PTH and RANKL signaling in bone remodeling may provide new insights into the pathogenesis of osteoporosis and identify novel targets for intervention.

In addition to these specific signaling pathways, there is growing recognition of the importance of cellular senescence and aging in the development of osteoporosis. Studies have shown that senescent cells accumulate in bone with age and contribute to the deterioration of bone microarchitecture and increased fracture risk (Pignolo et al., 2021). Targeting senescent cells through pharmacological or genetic approaches has emerged as a potential strategy for preventing age-related bone loss and osteoporosis (Föger-Samwald et al., 2022). Integrating insights from the biology of aging with our understanding of RANKL signaling may help to develop more comprehensive and effective approaches to osteoporosis prevention and treatment.

### Future directions and clinical implications

Our bibliometric analysis reveals several key trends that suggest promising directions for both research and clinical practice in osteoporosis management. In therapeutic development, the recent surge in RANKL-focused research (citation burst: 5.04, 2021–2024) alongside studies of cell differentiation suggests opportunities for next-generation treatments (Huang et al., 2022; Udagawa et al., 2021). Future therapeutic strategies should explore dual-action agents targeting both RANKL and newly identified molecular pathways, as well as tissue-specific RANKL modulators that reduce systemic effects (Patel and Saxena, 2025). Novel delivery systems and combination therapies integrating RANKL inhibition with anabolic agents represent particularly promising avenues for investigation.

The evolution of research focus from broad population studies to more targeted investigations signals an important shift toward personalized treatment approaches (D'Onofrio et al., 2022). This includes the development of genetic and molecular biomarkers for better predicting treatment response and identifying patient subgroups most likely to benefit from specific interventions (Yang et al., 2020; Kim B. J. et al., 2020). The integration of artificial intelligence for risk assessment and treatment optimization may further enhance our ability to customize treatment protocols based on individual patient characteristics (Lis-Studniarska et al., 2023; Wu and Dai, 2024).

Research trends highlight the need for improved clinical strategies in everyday practice. This encompasses the development of more sophisticated fracture risk assessment tools that go beyond bone mineral density measurements, along with standardized protocols for monitoring treatment response (Kim et al., 2019; Lorentzon et al., 2019). The growing understanding of RANKL’s extra-skeletal effects necessitates their integration into treatment decisions, supported by enhanced follow-up strategies for long-term therapy management (El-Gazzar and Högler, 2021).

Economic considerations will play a crucial role in implementing these advances. This requires robust cost-effectiveness studies of new therapeutic strategies and the development of value-based treatment algorithms (Liu and Xiao, 2023). Understanding the long-term economic impacts of various intervention strategies and analyzing healthcare resource utilization patterns will be essential for sustainable implementation.

### Strengths and limitations

This analysis presents a comprehensive summary of RANKL and osteoporotic fracture research over the last 24 years and provides insights into overarching long-term trends and changes in research direction. This study offers an in-depth view of the evolution of the field by calculating several bibliometric indicators (publications, citations, and collaborations) and applying sophisticated analyses such as keyword bursts. Nevertheless, a few limitations must be noted when interpreting these results. Firstly, the analysis was restricted to publications included in Web of Science database, which may overlook some relevant research, especially those from non-English languages. This may have led to the exclusion of relevant studies published in other languages, particularly those from non-English-speaking countries with significant research output in the field of osteoporosis and RANKL biology. Secondly, citation-based metrics can provide useful information, but they do not always capture the quality or significance of research. They can be influenced by self-citations, may disproportionately favor older publications that have had more time to accumulate citations, and can be skewed by a small number of highly-cited papers that may not necessarily represent the most important advances in the field. Thirdly, although the keyword burst analysis provided new insights, it may be affected by changes in terminology across time and may therefore fail to reflect the more subtle shifts in research focus.

## Conclusion

Over the past 2 decades (2001–2024), research interest in RANKL and osteoporotic fractures has shown consistent growth, with a 10.53% annual publication increase. Understanding the role of RANKL in bone metabolism continues to evolve, with the research focus shifting from basic biological mechanisms through clinical applications to a renewed interest in fundamental RANKL biology with new perspectives. This study comprehensively analyzed global research advancements in RANKL and osteoporotic fractures, and identified future research hotspots, including cell-type specific RANKL functions, the development of predictive biomarkers, and the optimization of combination therapeutic strategies.

The analysis revealed three key research priorities for future investigation. Understanding RANKL’s broader physiological roles emerges as a critical research direction, particularly given the recent surge in cellular differentiation studies. Clinical research priorities should focus on developing better predictive markers for treatment response and optimizing treatment strategies. The emerging focus on personalized medicine approaches suggests a need for studies identifying patient-specific factors that influence RANKL-targeted therapy outcomes.

As the global burden of osteoporosis continues to grow, addressing these research priorities will be crucial for advancing our understanding and treatment of bone disorders. Future studies on RANKL may aim to achieve more targeted therapeutic approaches, thereby reducing systemic effects and improving treatment efficacy. This analysis aims to guide the field toward more precise and personalized treatment approaches, providing scientific evidence and new therapeutic strategies for the treatment of osteoporosis and its related bone disorders. The strong international collaboration networks identified in our analysis provide a foundation for these future research efforts.

## Data Availability

The original contributions presented in the study are included in the article/Supplementary Material, further inquiries can be directed to the corresponding authors.

## References

[B1] Aria M. Cuccurullo C. (2017). bibliometrix: an R-tool for comprehensive science mapping analysis. J. Inf. 11 (4), 959–975. 10.1016/j.joi.2017.08.007

[B2] Armas L. A. Recker R. R. (2012). Pathophysiology of osteoporosis: new mechanistic insights. Endocrinol. Metabolism Clin. 41 (3), 475–486. 10.1016/j.ecl.2012.04.006 22877425

[B3] Ball R. (2017). An introduction to bibliometrics: new development and trends. Chandos Publishing. 10.1016/C2016-0-03695-1

[B4] Bertoli-Barsotti L. Lando T. (2017). A theoretical model of the relationship between the h-index and other simple citation indicators. Scientometrics 111, 1415–1448. 10.1007/s11192-017-2351-9 28596626 PMC5438441

[B5] Bone H. G. Wagman R. B. Brandi M. L. Brown J. P. Chapurlat R. Cummings S. R. (2017). 10 years of denosumab treatment in postmenopausal women with osteoporosis: results from the phase 3 randomised FREEDOM trial and open-label extension. lancet Diabetes and Endocrinol. 5 (7), 513–523. 10.1016/S2213-8587(17)30138-9 28546097

[B6] Bonewald L. F. (2011). The amazing osteocyte. J. bone mineral Res. 26 (2), 229–238. 10.1002/jbmr.320 PMC3179345 21254230

[B7] Boyce B. F. Xing L. (2007). Biology of RANK, RANKL, and osteoprotegerin. Arthritis Res. and Ther. 9, 1–7. 10.1186/ar2165 PMC1924516 17634140

[B8] Chen C. (2006). CiteSpace II: detecting and visualizing emerging trends and transient patterns in scientific literature. J. Am. Soc. Inf. Sci. Technol. 57 (3), 359–377. 10.1002/asi.20317

[B9] Chen X. Wang Z. Duan N. Zhu G. Schwarz E. M. Xie C. (2018). Osteoblast–osteoclast interactions. Connect. tissue Res. 59 (2), 99–107. 10.1080/03008207.2017.1290085 28324674 PMC5612831

[B10] Collin-Osdoby P. Rothe L. Anderson F. Nelson M. Maloney W. Osdoby P. (2001). Receptor activator of NF-κB and osteoprotegerin expression by human microvascular endothelial cells, regulation by inflammatory cytokines, and role in human osteoclastogenesis. J. Biol. Chem. 276 (23), 20659–20672. 10.1074/jbc.M010153200 11274143

[B11] D'Onofrio B. di Lernia M. De Stefano L. Bugatti S. Montecucco C. Bogliolo L. (2022). Personalized therapeutic strategies in the management of osteoporosis in patients with autoantibody-positive rheumatoid arthritis. J. Clin. Med. 11 (9), 2341. 10.3390/jcm11092341 35566466 PMC9104810

[B12] El-Gazzar A. Högler W. (2021). Mechanisms of bone fragility: from osteogenesis imperfecta to secondary osteoporosis. Int. J. Mol. Sci. 22 (2), 625. 10.3390/ijms22020625 33435159 PMC7826666

[B13] Estell E. G. Rosen C. J. (2021). Emerging insights into the comparative effectiveness of anabolic therapies for osteoporosis. Nat. Rev. Endocrinol. 17 (1), 31–46. 10.1038/s41574-020-00426-5 33149262

[B14] Estrada K. Styrkarsdottir U. Evangelou E. Hsu Y.-H. Duncan E. L. Ntzani E. E. (2012). Genome-wide meta-analysis identifies 56 bone mineral density loci and reveals 14 loci associated with risk of fracture. Nat. Genet. 44 (5), 491–501. 10.1038/ng.2249 22504420 PMC3338864

[B15] Föger-Samwald U. Kerschan-Schindl K. Butylina M. Pietschmann P. (2022). Age related osteoporosis: targeting cellular senescence. Int. J. Mol. Sci. 23 (5), 2701. 10.3390/ijms23052701 35269841 PMC8910503

[B16] Gennari L. Merlotti D. Falchetti A. Eller Vainicher C. Cosso R. Chiodini I. (2020). Emerging therapeutic targets for osteoporosis. Expert Opin. Ther. Targets 24 (2), 115–130. 10.1080/14728222.2020.1726889 32050822

[B17] Gould K. A. (2023). Journal citation reports 2023: understanding bibliometric data. LWW. 10.1097/DCC.0000000000000603 37523720

[B18] Hirsch J. E. (2005). An index to quantify an individual's scientific research output. Proc. Natl. Acad. Sci. 102 (46), 16569–16572. 10.1073/pnas.0507655102 16275915 PMC1283832

[B19] Hooshiar S. H. Tobeiha M. Jafarnejad S. (2022). Soy isoflavones and bone health: focus on the RANKL/RANK/OPG pathway. Biomed. Res. Int. 2022, 8862278. 10.1155/2022/8862278 36330454 PMC9626210

[B20] Huang D. Zhao C. Li R. Chen B. Zhang Y. Sun Z. (2022). Identification of a binding site on soluble RANKL that can be targeted to inhibit soluble RANK-RANKL interactions and treat osteoporosis. Nat. Commun. 13 (1), 5338. 10.1038/s41467-022-33006-4 36097003 PMC9468151

[B21] Infante M. Fabi A. Cognetti F. Gorini S. Caprio M. Fabbri A. (2019). RANKL/RANK/OPG system beyond bone remodeling: involvement in breast cancer and clinical perspectives. J. Exp. and Clin. Cancer Res. 38, 12–18. 10.1186/s13046-018-1001-2 30621730 PMC6325760

[B22] Kanis J. A. Harvey N. C. Johansson H. Liu E. Vandenput L. Lorentzon M. (2020). A decade of FRAX: how has it changed the management of osteoporosis? Aging Clin. Exp. Res. 32, 187–196. 10.1007/s40520-019-01432-y 32043227

[B23] Khosla S. Monroe D. G. (2018). Regulation of bone metabolism by sex steroids. Cold Spring Harb. Perspect. Med. 8 (1), a031211. 10.1101/cshperspect.a031211 28710257 PMC5749141

[B24] Kim B.-J. Lee S. H. Koh J.-M. (2020a). Potential biomarkers to improve the prediction of osteoporotic fractures. Endocrinol. Metabolism 35 (1), 55–63. 10.3803/EnM.2020.35.1.55 PMC7090300 32207264

[B25] Kim B. J. Lee S. H. Koh J. M. (2020b). Potential biomarkers to improve the prediction of osteoporotic fractures. Endocrinol. Metab. Seoul. 35 (1), 55–63. 10.3803/EnM.2020.35.1.55 32207264 PMC7090300

[B26] Kim B. Y. Kim H. A. Jung J. Y. Choi S. T. Kim J. M. Kim S. H. (2019). Clinical impact of the fracture risk assessment tool on the treatment decision for osteoporosis in patients with knee osteoarthritis: a multicenter comparative study of the fracture risk assessment tool and world health organization criteria. J. Clin. Med. 8 (7), 918. 10.3390/jcm8070918 31248035 PMC6678257

[B27] Kim S.-J. Lee D.-W. (2024). Publication trends in osteoporosis treatment: a 20-year bibliometric analysis. J. Bone Metabolism 31 (2), 90–100. 10.11005/jbm.2024.31.2.90 PMC11184155 38886967

[B28] Kumar D. Singh S. K. Sharma Y. (2021). Osteoporosis: a current update on globally epidemic asymptomatic disorder. Curr. Res. Trends Med. Sci. Technology2021, 129.

[B29] LeBoff M. S. Greenspan S. Insogna K. Lewiecki E. Saag K. Singer A. (2022). The clinician’s guide to prevention and treatment of osteoporosis. Osteoporos. Int. 33 (10), 2049–2102. 10.1007/s00198-021-05900-y 35478046 PMC9546973

[B30] Li D. Ou J. Zeng Y. Hou L. Yuan Y. Luo Z. (2023). Bibliometric study on clinical research of osteoporosis in adolescents. Front. Public Health 11, 1041360. 10.3389/fpubh.2023.1041360 36908434 PMC9992876

[B31] Lis-Studniarska D. Lipnicka M. Studniarski M. Irzmański R. (2023). Applications of artificial intelligence methods for the prediction of osteoporotic fractures. Life (Basel) 13 (8), 1738. 10.3390/life13081738 37629595 PMC10455761

[B32] Liu Z. Xiao L. (2023). Toward a value-based therapy recommendation model. Healthc. (Basel) 11 (16), 2362. 10.3390/healthcare11162362 PMC10454734 37628559

[B33] Lorentzon M. Branco J. Brandi M. L. Bruyère O. Chapurlat R. Cooper C. (2019). Algorithm for the use of biochemical markers of bone turnover in the diagnosis, assessment and follow-up of treatment for osteoporosis. Adv. Ther. 36 (10), 2811–2824. 10.1007/s12325-019-01063-9 31440982 PMC6822833

[B34] Marini F. Giusti F. Palmini G. Brandi M. L. (2023). Role of Wnt signaling and sclerostin in bone and as therapeutic targets in skeletal disorders. Osteoporos. Int. 34 (2), 213–238. 10.1007/s00198-022-06523-7 35982318

[B35] Matsumoto T. Endo I. (2021). RANKL as a target for the treatment of osteoporosis. J. bone mineral metabolism 39, 91–105. 10.1007/s00774-020-01153-7 33057808

[B36] Novak D. Batko M. Zezula P. (2011). Metric index: an efficient and scalable solution for precise and approximate similarity search. Inf. Syst. 36 (4), 721–733. 10.1016/j.is.2010.10.002

[B37] Patel D. Saxena B. (2025). Decoding osteoporosis: understanding the disease, exploring current and new therapies and emerging targets. J. Orthop. Rep. 4 (4), 100472. 10.1016/j.jorep.2024.100472

[B38] Pignolo R. J. Law S. F. Chandra A. (2021). Bone aging, cellular senescence, and osteoporosis. JBMR Plus 5 (4), e10488. 10.1002/jbm4.10488 33869998 PMC8046105

[B39] Rendina-Ruedy E. Rosen C. J. (2022). Parathyroid hormone (PTH) regulation of metabolic homeostasis: an old dog teaches us new tricks. Mol. Metab. 60, 101480. 10.1016/j.molmet.2022.101480 35338013 PMC8980887

[B40] Rong Y. f. Liang X. Z. Jiang K. Jia H. F. Li H. Z. Lu B. W. (2024). Global trends in research of programmed cell Death in osteoporosis: a bibliometric and visualized analysis (2000–2023). Orthop. Surg. 16 (8), 1783–1800. 10.1111/os.14133 38923347 PMC11293941

[B41] Sakai T. (2023). Fracture risks and their mechanisms in atopic dermatitis, focusing on receptor activator of nuclear factor kappa-B ligand. Clin. Exp. Dermatology 48 (11), 1209–1213. 10.1093/ced/llad220 37379576

[B42] Salisbury L. (2020). Scopus citescore and clarivate journal citation reports. Charlest. Advis. 21 (4), 5–15. 10.5260/chara.21.4.5

[B43] Schreiber M. (2010). How to modify the g-index for multi-authored manuscripts. J. Inf. 4 (1), 42–54. 10.1016/j.joi.2009.06.003

[B44] Shen Y. Huang X. Wu J. Lin X. Zhou X. Zhu Z. (2022). The global burden of osteoporosis, low bone mass, and its related fracture in 204 countries and territories, 1990-2019. Front. Endocrinol. 13, 882241. 10.3389/fendo.2022.882241 PMC9165055 35669691

[B45] Song S. Guo Y. Yang Y. Fu D. (2022). Advances in pathogenesis and therapeutic strategies for osteoporosis. Pharmacol. Ther. 237, 108168. 10.1016/j.pharmthera.2022.108168 35283172

[B46] Styrkarsdottir U. Halldorsson B. V. Gretarsdottir S. Gudbjartsson D. F. Walters G. B. Ingvarsson T. (2008). Multiple genetic loci for bone mineral density and fractures. N. Engl. J. Med. 358 (22), 2355–2365. 10.1056/NEJMoa0801197 18445777

[B47] Udagawa N. Koide M. Nakamura M. Nakamichi Y. Yamashita T. Uehara S. (2021). Osteoclast differentiation by RANKL and OPG signaling pathways. J. Bone Min. Metab. 39 (1), 19–26. 10.1007/s00774-020-01162-6 33079279

[B48] Van Eck N. Waltman L. (2010). Software survey: VOSviewer, a computer program for bibliometric mapping. scientometrics 84 (2), 523–538. 10.1007/s11192-009-0146-3 20585380 PMC2883932

[B49] Vasiliadis E. S. Evangelopoulos D. S. Kaspiris A. Benetos I. S. Vlachos C. Pneumaticos S. G. (2022). The role of sclerostin in bone diseases. J. Clin. Med. 11 (3), 806. 10.3390/jcm11030806 35160258 PMC8836457

[B50] Wu A.-M. Bisignano C. James S. L. Abady G. G. Abedi A. Abu-Gharbieh E. (2021). Global, regional, and national burden of bone fractures in 204 countries and territories, 1990–2019: a systematic analysis from the Global Burden of Disease Study 2019. Lancet Healthy Longev. 2 (9), e580–e592. 10.1016/S2666-7568(21)00172-0 34723233 PMC8547262

[B51] Wu Q. Dai J. (2024). Enhanced osteoporotic fracture prediction in postmenopausal women using Bayesian optimization of machine learning models with genetic risk score. J. Bone Min. Res. 39 (4), 462–472. 10.1093/jbmr/zjae025 PMC11262147 38477741

[B52] Yang T. L. Shen H. Liu A. Dong S. S. Zhang L. Deng F. Y. (2020). A road map for understanding molecular and genetic determinants of osteoporosis. Nat. Rev. Endocrinol. 16 (2), 91–103. 10.1038/s41574-019-0282-7 31792439 PMC6980376

[B53] Yao Z. Getting S. J. Locke I. C. (2021). Regulation of TNF-induced osteoclast differentiation. Cells 11 (1), 132. 10.3390/cells11010132 35011694 PMC8750957

[B54] Zhang C. Song C. (2020). Combination therapy of PTH and antiresorptive drugs on osteoporosis: a review of treatment alternatives. Front. Pharmacol. 11, 607017. 10.3389/fphar.2020.607017 33584284 PMC7874063

[B55] Zhang Y. Liang J. Liu P. Wang Q. Liu L. Zhao H. (2022). The RANK/RANKL/OPG system and tumor bone metastasis: potential mechanisms and therapeutic strategies. Front. Endocrinol. 13, 1063815. 10.3389/fendo.2022.1063815 PMC9800780 36589815

[B56] Zhu S. Chen W. Masson A. Li Y. P. (2024). Cell signaling and transcriptional regulation of osteoblast lineage commitment, differentiation, bone formation, and homeostasis. Cell Discov. 10 (1), 71. 10.1038/s41421-024-00689-6 38956429 PMC11219878

