# Supplementary material for: Global knowledge mapping of receptor activator of nuclear factor kappa-B ligand in osteoporotic fractures: a bibliometric analysis (2001–2024)
Source: Front Mol Biosci. 2025 Mar 26;12:1545109. doi: 10.3389/fmolb.2025.1545109 (PMC11978631; doi:10.3389/fmolb.2025.1545109)
Supplement: Supplementary file 1 [file DataSheet1.docx]

**Table S1 Publication and Citation Profiles of Leading Countries**

| **Country** | **Articles** | **Freq** | **SCP** | **MCP** | **MCP_Ratio** | **TP** | **TP_rank** | **TC** | **TC_rank** | **Average Citations** |
| --- | --- | --- | --- | --- | --- | --- | --- | --- | --- | --- |
| CHINA | 60 | 0.28 | 52 | 8 | 0.133 | 200 | 1 | 1046 | 2 | 17.4 |
| USA | 26 | 0.121 | 14 | 12 | 0.462 | 153 | 2 | 1808 | 1 | 69.5 |
| KOREA | 21 | 0.098 | 18 | 3 | 0.143 | 85 | 3 | 339 | 6 | 16.1 |
| ITALY | 13 | 0.061 | 10 | 3 | 0.231 | 46 | 5 | 462 | 4 | 35.5 |
| GERMANY | 11 | 0.051 | 10 | 1 | 0.091 | 61 | 4 | 327 | 7 | 29.7 |
| SPAIN | 9 | 0.042 | 9 | 0 | 0 | 43 | 7 | 298 | 8 | 33.1 |
| JAPAN | 8 | 0.037 | 8 | 0 | 0 | 33 | 9 | 210 | 12 | 26.2 |
| BRAZIL | 5 | 0.023 | 4 | 1 | 0.2 | 17 | 13 | 114 | 15 | 22.8 |
| INDIA | 5 | 0.023 | 5 | 0 | 0 | 14 | 18 | 35 | 24 | 7 |
| UNITED KINGDOM | 5 | 0.023 | 1 | 4 | 0.8 | 46 | 6 | 114 | 17 | 22.8 |
| IRAN | 4 | 0.019 | 3 | 1 | 0.25 | 15 | 15 | 67 | 18 | 16.8 |
| SWITZERLAND | 4 | 0.019 | 1 | 3 | 0.75 | 11 | 23 | 249 | 10 | 62.2 |
| CANADA | 3 | 0.014 | 1 | 2 | 0.667 | 36 | 8 | 216 | 11 | 72 |
| DENMARK | 3 | 0.014 | 3 | 0 | 0 | 14 | 16 | 253 | 9 | 84.3 |
| EGYPT | 3 | 0.014 | 1 | 2 | 0.667 | 9 | 27 | 25 | 25 | 8.3 |
| GREECE | 3 | 0.014 | 2 | 1 | 0.333 | 14 | 17 | 114 | 16 | 38 |
| SLOVENIA | 3 | 0.014 | 3 | 0 | 0 | 13 | 19 | 173 | 14 | 57.7 |
| AUSTRALIA | 2 | 0.009 | 2 | 0 | 0 | 26 | 10 | 16 | 29 | 8 |
| BULGARIA | 2 | 0.009 | 1 | 1 | 0.5 | 5 | 32 | 6 | 36 | 3 |
| MEXICO | 2 | 0.009 | 1 | 1 | 0.5 | 19 | 12 | 9 | 33 | 4.5 |

Note(s): Articles: Publications of Corresponding Authors only. Freq: Frequence of Total Publications. SCP: Single Country publications. MCP: Multiple Country Publications. MCP_Ratio: Proportion of Multiple Country Publications. TP: Total Publications. TP_rank: Rank of Total Publications. TC: Total Citations. TC_rank: Rank of Total Citations. Average Citations: The average number of citations per publication.

**Table S2 Bibliometric Indicators of High-Impact Journals**

| **Journal** | **H_index** | **IF 2023** | **JCR 2023** | **TP** | **TP_rank** | **TC** | **TC_rank** | **PY_start** |
| --- | --- | --- | --- | --- | --- | --- | --- | --- |
| JOURNAL OF BONE AND MINERAL RESEARCH | 9 | 5.1 | 1 | 10 | 1 | 741 | 1 | 2002 |
| OSTEOPOROSIS INTERNATIONAL | 9 | 4.2 | 1 | 9 | 2 | 394 | 3 | 2005 |
| BONE | 8 | 3.5 | 2 | 8 | 3 | 491 | 2 | 2011 |
| CALCIFIED TISSUE INTERNATIONAL | 7 | 3.3 | 2 | 7 | 4 | 154 | 7 | 2005 |
| PLOS ONE | 6 | 2.9 | 1 | 7 | 5 | 101 | 14 | 2011 |
| FRONTIERS IN PHARMACOLOGY | 5 | 4.4 | 1 | 5 | 7 | 27 | 45 | 2018 |
| JOURNAL OF BONE AND MINERAL METABOLISM | 4 | 2.4 | 3 | 4 | 10 | 72 | 18 | 2007 |
| JOURNAL OF ORTHOPAEDIC RESEARCH | 4 | 2.1 | 2 | 5 | 8 | 49 | 26 | 2014 |
| MOLECULES | 4 | 4.2 | 2 | 6 | 6 | 25 | 51 | 2018 |
| BIOMED RESEARCH INTERNATIONAL | 3 | 2.6 | 3 | 3 | 11 | 14 | 95 | 2016 |
| BONE RESEARCH | 3 | 14.3 | 1 | 4 | 9 | 15 | 89 | 2017 |
| JOURNAL OF STEROID BIOCHEMISTRY AND MOLECULAR BIOLOGY | 3 | 2.7 | 3 | 3 | 14 | 22 | 58 | 2009 |
| AGING-US | 2 | 3.9 | 2 | 2 | 16 | 3 | 410 | 2020 |
| AMERICAN JOURNAL OF TRANSLATIONAL RESEARCH | 2 | 1.7 | 3 | 2 | 17 | 1 | 873 | 2018 |
| BIOMATERIALS | 2 | 12.8 | 1 | 2 | 18 | 67 | 21 | 2018 |
| BIOMOLECULES | 2 | 4.8 | 1 | 2 | 20 | 2 | 578 | 2020 |
| BMC MUSCULOSKELETAL DISORDERS | 2 | 2.2 | 2 | 2 | 21 | 16 | 85 | 2013 |
| CELL DEATH & DISEASE | 2 | 8.1 | 1 | 2 | 22 | 12 | 116 | 2017 |
| EUROPEAN JOURNAL OF ENDOCRINOLOGY | 2 | 5.3 | 1 | 2 | 23 | 26 | 48 | 2010 |
| EXPERIMENTAL AND THERAPEUTIC MEDICINE | 2 | 2.4 | 3 | 2 | 24 | 15 | 90 | 2018 |

Note(s): H_index: The H-index of the journal, which measures both the productivity and citation impact of the publications. IF: Impact Factor, indicating the average number of citations to recent articles published in the journal. JCR_Quartile: The quartile ranking of the journal in the Journal Citation Reports, indicating the journal's ranking relative to others in the same field (Q1: top 25%, Q2: 25%-50%, Q3: 50%-75%, Q4: bottom 25%). TP: Total Publications. TP_rank: Rank of Total Publications. TC: Total Citations. TC_rank: Rank of Total Citations. PY_start: Publication Year Start, indicating the year the journal started publication.

**Table S3 Publication and Citation Profiles of High-Impact Authors**

| **Author** | **H_index** | **G-index** | **M-index** | **PY_start** | **TP** | **TP_Frac** | **TP_rank** | **TC** | **TC_rank** |
| --- | --- | --- | --- | --- | --- | --- | --- | --- | --- |
| EL KHASSAWNA THAQIF | 5 | 5 | 0.417 | 2013 | 5 | 0.28 | 2 | 153 | 9 |
| HEISS CHRISTIAN | 5 | 6 | 0.417 | 2013 | 6 | 0.36 | 1 | 153 | 9 |
| ALT VOLKER | 4 | 5 | 0.333 | 2013 | 5 | 0.31 | 2 | 142 | 11 |
| MARC JANJA | 4 | 4 | 0.222 | 2007 | 4 | 0.71 | 4 | 1078 | 2 |
| PREZELJ JANEZ | 4 | 4 | 0.222 | 2007 | 4 | 0.71 | 4 | 1078 | 2 |
| CHRISTIANSEN CLAUS | 3 | 3 | 0.158 | 2006 | 3 | 0.31 | 10 | 1368 | 1 |
| FERRARI SERGE | 3 | 3 | 0.273 | 2014 | 3 | 0.4 | 10 | 246 | 7 |
| GINER MERCE | 3 | 3 | 0.188 | 2009 | 3 | 0.5 | 10 | 83 | 18 |
| KAMPSCHULTE MARIAN | 3 | 3 | 0.25 | 2013 | 3 | 0.16 | 10 | 123 | 13 |
| LIPS KATRIN S. | 3 | 4 | 0.333 | 2016 | 4 | 0.24 | 4 | 106 | 14 |
| MENCEJ-BEDRAC SIMONA | 3 | 3 | 0.2 | 2010 | 3 | 0.54 | 10 | 955 | 5 |
| PEREZ-CANO RAMON | 3 | 3 | 0.188 | 2009 | 3 | 0.5 | 10 | 83 | 18 |
| RAY SEEMUN | 3 | 4 | 0.333 | 2016 | 4 | 0.25 | 4 | 106 | 14 |
| RIANCHO JOSE A. | 3 | 3 | 0.214 | 2011 | 3 | 0.3 | 10 | 1022 | 4 |
| ROHNKE MARCUS | 3 | 4 | 0.333 | 2016 | 4 | 0.25 | 4 | 106 | 14 |
| SCHNETTLER REINHARD | 3 | 3 | 0.25 | 2013 | 3 | 0.17 | 10 | 131 | 12 |
| STOLINA MARINA | 3 | 3 | 0.214 | 2011 | 3 | 0.37 | 10 | 159 | 8 |
| THORMANN ULRICH | 3 | 4 | 0.333 | 2016 | 4 | 0.25 | 4 | 106 | 14 |
| ADACHI J. D. | 2 | 2 | 0.154 | 2012 | 2 | 0.32 | 19 | 260 | 6 |
| ADAMS JUDITH E. | 2 | 2 | 0.133 | 2010 | 2 | 0.09 | 19 | 74 | 20 |

Note(s): H-index: The index measures both the productivity and citation impact of the author’s publications. G-index: The index evaluates the cumulative impact of the author’s most highly cited publications. M-index: The index is the H-index divided by the number of years since the authors’s first publication. PY_start: Publication Year Start, indicating the year the journal started publication. TP: Total Publications. TP_Frac: Total Publications Fractionalized. TP_rank: Rank of Total Publications. TC: Total Citations. TC_rank: Rank of Total Citations.

**Table S4. The Top 50 Highly Cited Articles.**

| **Paper** | **DOI** | **Total Citations** | **TC per Year** | **Normalized TC** |
| --- | --- | --- | --- | --- |
| ESTRADA K, 2012, NAT GENET | 10.1038/ng.2249 | 905 | 69.62 | 6.74 |
| STYRKARSDOTTIR U, 2008, NEW ENGL J MED | 10.1056/NEJMoa0801197 | 434 | 25.53 | 3.59 |
| COLLIN-OSDOBY P, 2001, J BIOL CHEM | 10.1074/jbc.M010153200 | 328 | 13.67 | 1.00 |
| FAN Y, 2017, CELL METAB | 10.1016/j.cmet.2017.01.001 | 275 | 34.38 | 4.80 |
| TAKESHITA S, 2002, NAT MED | 10.1038/nm752 | 209 | 9.09 | 1.11 |
| HANLEY DA, 2012, INT J CLIN PRACT | 10.1111/ijcp.12022 | 204 | 15.69 | 1.52 |
| BONNET N, 2019, J CLIN INVEST | 10.1172/JCI125915 | 175 | 29.17 | 3.92 |
| REID IR, 2010, J BONE MINER RES | 10.1002/jbmr.149 | 175 | 11.67 | 3.03 |
| LANGDAHL BL, 2002, J BONE MINER RES | 10.1359/jbmr.2002.17.7.1245 | 166 | 7.22 | 0.89 |
| HAUSSLER MR, 2010, J STEROID BIOCHEM | 10.1016/j.jsbmb.2010.03.019 | 132 | 8.80 | 2.28 |
| MAZZIOTTI G, 2012, ENDOCRINE | 10.1007/s12020-011-9570-2 | 128 | 9.85 | 0.95 |
| PIEMONTESE M, 2017, JCI INSIGHT | 10.1172/jci.insight.93771 | 126 | 15.75 | 2.20 |
| LOGAR DB, 2007, J BONE MINER METAB | 10.1007/s00774-007-0753-0 | 123 | 6.83 | 2.49 |
| ZHANG J, 2017, BONE RES | 10.1038/boneres.2016.56 | 117 | 14.63 | 2.04 |
| PENG SL, 2011, BONE | 10.1016/j.bone.2011.08.031 | 114 | 8.14 | 2.84 |
| LUEGMAYR E, 2004, CELL DEATH DIFFER | 10.1038/sj.cdd.4401399 | 113 | 5.38 | 1.64 |
| POLZER K, 2010, ANN RHEUM DIS | 10.1136/ard.2008.104786 | 101 | 6.73 | 1.75 |
| TOMIMORI Y, 2009, J BONE MINER RES | 10.1359/JBMR.090217 | 96 | 6.00 | 1.48 |
| DELGADO-CALLE J, 2012, EPIGENETICS-US | 10.4161/epi.7.1.18753 | 93 | 7.15 | 0.69 |
| RANA T, 2012, FREE RADICAL BIO MED | 10.1016/j.freeradbiomed.2012.10.536 | 89 | 6.85 | 0.66 |
| OMINSKY MS, 2017, BONE | 10.1016/j.bone.2016.10.019 | 81 | 10.13 | 1.41 |
| RAY S, 2018, BIOMATERIALS | 10.1016/j.biomaterials.2017.11.049 | 76 | 10.86 | 2.77 |
| LEE NH, 2021, BIOMATERIALS | 10.1016/j.biomaterials.2021.121025 | 75 | 18.75 | 5.08 |
| OMINSKY MS, 2011, BONE | 10.1016/j.bone.2011.04.001 | 73 | 5.21 | 1.82 |
| ZHAO HY, 2005, OSTEOPOROSIS INT | 10.1007/s00198-005-1865-9 | 70 | 3.50 | 1.27 |
| SANZ-SALVADOR L, 2015, EUR J ENDOCRINOL | 10.1530/EJE-14-0424 | 66 | 6.60 | 2.06 |
| WON HY, 2011, PLOS ONE | 10.1371/journal.pone.0018168 | 64 | 4.57 | 1.59 |
| TAN EM, 2017, J BONE MINER RES | 10.1002/jbmr.3031 | 62 | 7.75 | 1.08 |
| PINO AM, 2010, J BONE MINER RES | 10.1359/jbmr.090802 | 60 | 4.00 | 1.04 |
| ROSHANDEL D, 2010, J BONE MINER RES | 10.1002/jbmr.78 | 59 | 3.93 | 1.02 |
| ABDALLAH BM, 2005, CALCIFIED TISSUE INT | 10.1007/s00223-004-0074-4 | 58 | 2.90 | 1.05 |
| LIU JM, 2010, J CLIN ENDOCR METAB | 10.1210/jc.2009-2768 | 58 | 3.87 | 1.00 |
| FERRARI S, 2015, OSTEOPOROSIS INT | 10.1007/s00198-015-3179-x | 56 | 5.60 | 1.75 |
| RINOTAS V, 2014, J BONE MINER RES | 10.1002/jbmr.2112 | 55 | 5.00 | 1.95 |
| JING D, 2013, PLOS ONE | 10.1371/journal.pone.0079377 | 55 | 4.58 | 2.56 |
| CHEUNG WH, 2011, ULTRASOUND MED BIOL | 10.1016/j.ultrasmedbio.2010.11.016 | 54 | 3.86 | 1.34 |
| LI YF, 2012, OSTEOPOROSIS INT | 10.1007/s00198-011-1751-6 | 49 | 3.77 | 0.36 |
| BOUDIN E, 2016, MOL CELL ENDOCRINOL | 10.1016/j.mce.2015.12.021 | 49 | 5.44 | 2.49 |
| TOHIDI M, 2012, BONE | 10.1016/j.bone.2012.08.117 | 48 | 3.69 | 0.36 |
| ZHANG ZG, 2014, INT J MOL SCI | 10.3390/ijms150917130 | 46 | 4.18 | 1.63 |
| LARSSON S, 2014, ARCH ORTHOP TRAUM SU | 10.1007/s00402-012-1558-8 | 46 | 4.18 | 1.63 |
| LIAN WS, 2019, CELL DEATH DIS | 10.1038/s41419-019-1942-1 | 45 | 7.50 | 1.01 |
| CATALANO A, 2018, BONE | 10.1016/j.bone.2018.07.010 | 42 | 6.00 | 1.53 |
| FESTUCCIA F, 2017, J NEPHROL | 10.1007/s40620-016-0334-1 | 41 | 5.13 | 0.72 |
| CHUNG SL, 2014, J ORTHOP RES | 10.1002/jor.22715 | 40 | 3.64 | 1.42 |
| SCIMECA M, 2017, CELL DEATH DIS | 10.1038/cddis.2017.514 | 39 | 4.88 | 0.68 |
| XIE F, 2005, BIOL PHARM BULL | 10.1248/bpb.28.1879 | 38 | 1.90 | 0.69 |
| WEN BH, 2018, EXP THER MED | 10.3892/etm.2018.6043 | 38 | 5.43 | 1.38 |
| CAETANO-LOPES J, 2011, PLOS ONE | 10.1371/journal.pone.0016947 | 38 | 2.71 | 0.95 |
| D'AMELIO P, 2011, OSTEOPOROSIS INT | 10.1007/s00198-010-1496-7 | 36 | 2.57 | 0.90 |

Note(s): TC: Total Citations.
